# Practical Observations on Ship Fever as It Prevailed in Philadelphia and Its Environs, in the Months of June, July, and August, 1847

**Published:** 1847-09

**Authors:** Laurence Turnbull

**Affiliations:** 385 Spruce street, Philadelphia


### *Practical Observations on Ship Fever as it prevailed in Philadelphia and its environs, in the months of June, July, and August,* 1847. By Laurence Turnbull, M. D.

This disease prevails at the present time to some extent as an epidemic in our city and suburbs. During the last three months, there have been over three hundred cases admitted into the almshouse, and a corresponding number under the care of physicians resident in the city. Having had a number of cases in charge, I have taken this method of making known my views of its nature and treatment, hoping my endeavors may add something to the general stock of knowledge concerning it.

The first great cause of this fever may be traced to the starving state of the population of Ireland; with the subsequent crowded condition of our immigrant ships, without a proper attention to cleanliness and ventilation on the part of their commanders; thus causing the generation of an animal effluvium, which, contaminating the surrounding atmosphere, is received into the lungs, thence proceeding into the circulation. The extreme series of organic capillaries being thus irritated, occasions a subversion of the existing mode of action, through which the different secretions and functions are diminished, increased, or modified.

This disease attacks all ages, from the infant to the old man, and not the poor immigrant only, but the residents of our city, passing by means of this contagious effluvium into the lungs of all constantly exposed to its baneful influence. There cannot be a doubt concerning its contagious nature, as nine of the most severe cases out of twenty-one, in my own practice, were those residing in the same houses with the sick, none of them having been on board ship, but citizens for some time.

The period which elapses before the disease shows itself averages from one to two weeks, but in most cases it makes its appearance in a few days after leaving the vessel.

Nearly every ship which enters our port brings a greater or less number of invalids, some having suffered for twenty-six and twenty-eight days, without proper nourishment or medical attendance. In many instances, the number of passengers in the Liverpool packets to New York, has exceeded five hundred, and the regulations of those ships obliged them to leave their berths between the hours of three and four, A. M., for the purpose of cleansing them, so much water being used in this process as to leave them in a damp and unhealthy condition for many hours. The water for culinary purposes was not distributed until nine, so that the strong and active alone procured a meal before twelve or one, P. M. To insure personal cleanliness for those who were not so disposed, a barrel of water was provided and a portion of it poured over the poor creatures, who were not even fortified by proper food. It cannot therefore be a matter of astonishment that a number should be sick on the voyage, and a still larger number fit only for the hospital on their arrival, having had within the vessel all the elements to generate typhus fever. First, their crowded condition; secondly, their want of proper rest; thirdly, the want of proper nourishment; and fourthly, the damp condition of their sleeping and eating apartments.

The authorities now remove the sick either into the Lazaretto or the fever hospital at Bush Hill, which was opened for the reception of patients on the ninth of July, under the charge of Drs. L. W. Knight and William Ashley; one hundred and eighty cases having been admitted from that time up to the eighteenth of August. Of that number thirteen have died, thirty have been discharged cured, and one hundred and thirty-seven remain under treatment.

Of the thirteen cases which terminated fatally, three were in a dying state when admitted, and three died of purpura hæmorrhagica, marasmus and phthisis pulmonalis, thus considerably reducing the number of deaths by fever, and showing that the mortality is small in comparison with the accounts received from Canada and New York.

The situation of the hospital is very favourable, being light, dry, and well ventilated. The only objection is the want of space, obliging them to crowd the wards, so that dysentery or diarrhœa occurring as a complication, it is apt to assume a malignant form.

The premonitory symptoms are as follows: Slight chill, skin hot and dry, nausea, and in some cases vomiting, muscular pains in the back and limbs, with intense pain in the frontal region, increasing from the first to the sixth day. Tongue of a whitish color over the surface, tip of bright red; pulse averaging from eighty-six to one hundred and twenty, with bowels costive.

These symptoms continue for several days, when the skin becomes of a dusky red, the eyes are injected, tongue covered with a thick yellowish, brown, or black paste; teeth covered with sordes; acute pain over the epigastric region, with skin covered with a petechial eruption coming out from the sixth to the tenth day. Delirium now supervenes, with congestion of the lungs, brain or bronchial tubes, generally accompanied by deafness, with spasmodic twitchings of the muscles, and in some cases dysentery and profuse sweats, not of a critical nature.

These symptoms either increase or diminish towards the thirteenth or fourteenth day, or may terminate in death from the twentieth to the thirtieth day, the liability to relapse being very great in all cases.

I have found the following symptoms favorable when occurring about the fourteenth or twenty-first day: tongue becoming moist, first at edges; slight salivation; pulse fuller and slower, with tongue not tremulous when protruded. Unfavorable symptoms: great muscular debility; decubitus, or gliding down to the bottom of the bed, picking at the clothes, or catching at imaginary objects.

*Treatment.*—In the first stage a gentle aperient of oleum ricini or mild neutral salt. Second stage; to diminish the fever by freely sponging the body with tepid water, with the free use of ice or neutral mixture. Thirdly; to subdue local inflammation by cups or leeches, and by applying cold by means of ice to the head, or blister to the nape of the neck, with small doses of hydrargyrum cum cretâ and pulvis rhei. Lastly; to give quiniæ sulphas in pills of one grain three times a day, with from f℥iv. to f℥vi. of port wine.

By following the above course of treatment, bearing in mind ventilation and cleanliness, with a simple farinaceous diet, out of twenty-one cases I have lost but two, and regretted much that in both instances there was no opportunity for a post mortem examination.

385 *Spruce street, Philadelphia.*

